# Correction: A Kolmogorov-Smirnov test for the molecular clock based on Bayesian ensembles of phylogenies

**DOI:** 10.1371/journal.pone.0193633

**Published:** 2018-02-23

**Authors:** The PLOS ONE Staff

In Eq (1), the denominator appears incorrectly. The publisher apologizes for this error. Please view the correct equation here:

$$FN\left(x\right)=\frac{number\;of\;elements\;in\;the\;sample\;which\;are\leq x}{N}$$ (1)

In the Program availability section, the URL provided is incorrect. The correct URL is github.com/FernandoMarcon/PKS-Test.

## References

[1] Antoneli F, Passos FM, Lopes LR, Briones MRS (2018) A Kolmogorov-Smirnov test for the molecular clock based on Bayesian ensembles of phylogenies. PLoS ONE 13(1): e0190826 https://doi.org/10.1371/journal.pone.0190826 29300759 10.1371/journal.pone.0190826 PMC5754089

